# Chemical Structure and Thermal Properties versus Accelerated Aging of Bio-Based Poly(ether-urethanes) with Modified Hard Segments

**DOI:** 10.3390/molecules29153585

**Published:** 2024-07-30

**Authors:** Julia Godlewska, Joanna Smorawska, Ewa Głowińska

**Affiliations:** Department of Polymers Technology, Faculty of Chemistry, Gdansk University of Technology, 11/12 Gabriel Narutowicza Street, 80-233 Gdansk, Poland

**Keywords:** accelerated aging, bio-based poly(ether-urethanes), phase separation, thermal stability

## Abstract

Aging of polymers is a natural process that occurs during their usage and storage. Predicting the lifetime of polymers is a crucial aspect that should be considered at the design stage. In this paper, a series of bio-based thermoplastic poly(ether-urethane) elastomers (bio-TPUs) with modified hard segments were synthesized and investigated to understand the structural and property changes triggered by accelerated aging. The bio-TPUs were synthesized at an equimolar ratio of reagents using the prepolymer method with the use of bio-based poly(trimethylene ether) glycol, bio-based 1,3-propanediol, and hexamethylene diisocyanate or hexamethylene diisocyanate/partially bio-based diisocyanate mixtures. The polymerization reaction was catalyzed by dibutyltin dilaurate (DBTDL). The structural and property changes after accelerated aging under thermal and hydrothermal conditions were determined using Fourier transform infrared spectroscopy (FTIR), differential scanning calorimetry (DSC), thermogravimetric analysis (TGA), and dynamic mechanical thermal analysis (DMTA). Among other findings, it was observed that both the reference and aged bio-TPUs decomposed in two main stages and exhibited thermal stability up to approximately 300 °C. Based on the research conducted, it was found that accelerated aging impacts the supramolecular structure of TPUs.

## 1. Introduction

Aging of materials is an inevitable process in the life cycle of every product, leading to material fragmentation, loss of cohesion, and changes in properties. This process is unfavorable because it can result in the accumulation of microplastics in the environment [1]. By definition, aging occurs when the properties of a polymer change over time [2]. Moreover, aging is a complex process that occurs regardless of the polymer’s chemical composition. Polymer materials are susceptible to various factors that can cause their decomposition, such as thermal, chemical, biological, physical, or mechanical influences [3]. The degradation mechanism primarily depends on the chemical structure of the polymer and the aging factors involved. Typically, during the use of polymers, these factors act simultaneously. At the laboratory scale, it is possible to simulate different aging agents and investigate their impact on polymeric materials [3]. Nevertheless, it should be mentioned that laboratory simulations cannot fully replace the natural environment where polymers are used, but they can be helpful in determining the limitations and conditions of polymer applications.

It is well known that thermoplastic polyurethane elastomers (TPUs) belong to the large family of polyurethane materials. TPUs are characterized by a linear or slightly branched segmented structure, which makes them easy to process using methods typical for thermoplastic materials, such as extrusion or injection molding [4,5]. The properties of TPUs depend on the chemical structure of the monomers used for their synthesis, the molar ratio of the main components, as well as the synthesis method [6,7]. Furthermore, TPUs exhibit a segmented structure, meaning they are composed of hard (HS) and soft (SS) segments [8]. In addition to the presence of urethane linkages, the structure of TPUs includes other functional groups such as ester, ether, and urea, which contribute to the unique properties of these materials. This is why TPUs can be characterized by enhanced resistance to specific factors. Generally, it is predicted that poly(ether-urethanes) exhibit better resistance to hydrolysis compared to poly(ester-urethanes), although they are less stable under thermo-oxidative conditions [9,10]. The thermal degradation of polyurethanes is usually associated with thermo-oxidative processes resulting in the thermal dissociation of the chemical bonds in the macromolecules and/or the presence of free radicals [11]. According to the literature, this thermal aging causes the formation of free radicals, leading to an increase in the degree of network reticulation in thermoplastic polyurethane materials [12].

Due to the ability to modify the structure of TPU, the resistance of these polymers to different factors can vary [7,13]. Therefore, aging studies should typically be conducted before introducing these polymers for use. Such studies aim to simulate natural conditions such as temperature and water environment that occur during material usage. The structural changes can be observed through spectroscopy and thermal analyses. The examination of glass transition temperature (T_g_) can be considered an indicator revealing degradation of the material structure. In the case of hydrothermal aging, a decrease in T_g_ is associated with the plasticization phenomenon that occurs during the immersion period of the material [14].

Boubakri et al. investigated the commercially available polycaprolactone copolyester-based thermoplastic polyurethane elastomer immersed in distilled water at set temperatures of 25, 70, and 90 °C for moderate immersion times. They found that temperature and time affect the sorption properties and also lead to changes in the mechanical properties [14]. In another work, Boubakri et al. examined the impact of thermal aging on the tensile and creep behavior of TPUs based on polycaprolactone copolyester. Thermal aging was conducted at temperatures of 70 and 90 °C for over 270 days. This aging process caused changes in the appearance of TPU, a decrease in creep strain, and an increase in material rigidity. The authors explained these phenomena as being due to the decrease in the molecular mobility governed by the molecular rearrangements [12]. 

The thermal aging of the physicochemical properties of poly(ester-urethanes) obtained via the prepolymer method was investigated by Rosu et al. The materials underwent accelerated thermal aging at temperatures of 40 °C, 70 °C, 100 °C, and 130 °C up to 200 h. The authors observed changes in the material properties. Based on FTIR spectra, they found that urethane bond scission occurred during the thermal treatment [13]. Hong et al. also investigated the effect of thermal treatment on the properties and molecular changes of poly(ether-urethanes). Polyurethanes were prepared by the prepolymer method using poly(tetramethylene ether) glycol, toluene Diisocyanate, and 4,4′-methylene-bis-2-chloroaniline (MBOCA). Aging tests were performed at temperatures of 50, 60, 70, and 80 °C, with aging times of 32, 16, 8, and 4 days. Based on the conducted measurements, the authors suggested that changes in the hydrogen bonding and decomposition mechanism occurred in the hard segment region. In general, the tensile properties depended on the aging time [15]. 

The hygrothermal aging of poly(ether-urethanes) was investigated by Jana and Bhunia. The aging cycles involved exposing the materials to 74.9 °C and 10% humidity for 48 h and then 23.5 °C and 100% humidity for 48 h, followed by a decrease in temperature to −58 °C and 100% humidity for 8 h, and in the last cycle, an increase in temperature to 39 °C and 100% of humidity for 64 h. Samples were taken from the aging chamber after 720, 1440, and 2160 h of exposure. Marginal structural changes were revealed by Fourier transform spectroscopy (FTIR) and differential scanning calorimetry (DSC). After 2160 h, hygrothermal aging caused a slight decrease in tensile strength [16].

The novelty of this work lies in studying the influence of accelerated thermal and hydrothermal aging on bio-based thermoplastic poly(ether-urethane) elastomers (bio-TPUs). The aforementioned aging conditions and their impact on TPU properties might be revealed in the chemical structure and thermal properties, due to intermolecular structure reorganization.

## 2. Results and Discussion

### 2.1. Spectroscopic Analysis of Chemical Structure and Degree of Phase Separation in Bio-TPUs

**Figure 1:**
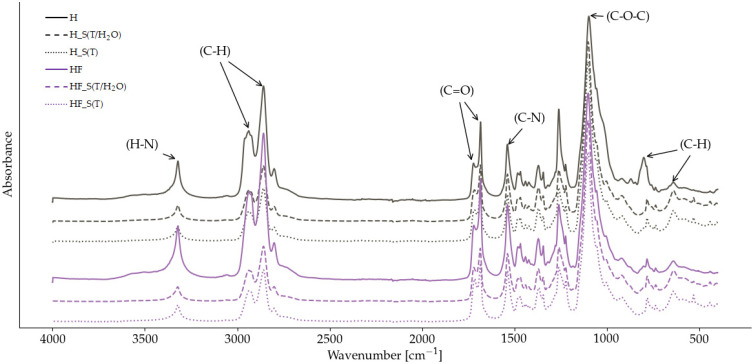
FTIR spectra of bio-TPUs before (H and HF) and after accelerated aging (H_S(T/H_2_O; H_S(T), HF_S(T/H_2_O; HF_S(T)).

Fourier transform infrared spectroscopy (FTIR) was used to analyze the chemical structure of bio-based thermoplastic poly(ether-urethane) elastomers and to investigate the impact of modifying the hard segments on the aging behavior of bio-TPUs under thermal and hydrothermal conditions. In Figure 1, the spectra of the reference materials (H), modified bio-TPU (HF), and both materials after accelerated aging are presented. Analyzing the spectra of the reference (H) and modified (HF) bio-TPUs, the characteristic vibrations of atom bonds in the urethane groups (NHC(O)O) were confirmed by the presence of the characteristic absorptions. Both bio-TPUs showed the absence of signals in the range of 2250–2270 cm^−1^, corresponding to the isocyanate groups, indicating the completion of the polymerization reaction. The stretching vibration of N-H bonds appeared at 3300 cm^−1^, while the carbonyl groups were detected in the wavenumber range of 1670–1730 cm^−1^ [17]. 

In the case of the carbonyl group, the multiplet peak was registered, indicating the presence of both free and hydrogen-bonded (H-bonded) carbonyls in the urethane structure. The nature of the polyether polyol was evident from the stretching vibrations ranging from 1135 to 1000 cm^−1^, originating from the ether group (C-O-C) [18]. Due to the aliphatic structure of bio-TPUs, the characteristic stretching and deformation vibrations of C-H bonds were observed in the wavenumber range from 3000 to 2900 cm^−1^, and below 1000 cm^−1^.

Aging under both studied conditions revealed scission of the urethane bonds in the FTIR spectra. A decrease in the peak intensities derived from the stretching vibrations of N-H (at 3300 cm^−1^), C-H (2800–3000 cm^−1^), and C=O (1660–1740 cm^−1^) was observed. A closer examination of the stretching vibration of the carbonyl group indicates changes in the content of the hydrogen bonds, suggesting changes in the supramolecular structure of TPUs. This implies that accelerated aging primarily affects hard segments, rather than soft segments. Notably, no significant changes were observed in the wavenumber range corresponding to the stretching vibration of the ether group.

Due to the importance of the interurethane hydrogen bonding for thermal properties and phase separation, FTIR spectra were used to calculate the degree of phase separation (DPS) based on the decomposition of the carbonyl peak. As mentioned earlier, the carbonyl group appeared as a multiplate peak, indicating the presence of both free and hydrogen bonded (H-bonded) carbonyls in the urethane. Furthermore, we were able to identify the positions of H-bonded carbonyl groups in the ordered and disordered phases. Therefore, the carbonyl peak was decomposed into four component peaks: H-bonded carbonyl in the ordered phase at ca. 1682 cm^−1^, H-bonded carbonyl in the disordered phase at around 1689 cm^−1^, free carbonyl in the ordered phase at about 1707 cm^−1^, and free carbonyl at the disordered phase at 1724 cm^−1^. A comparison with the literature revealed that the prepared bio-TPUs exhibited a lower DPS than previously described materials [18,19]. Analyzing Table 1, we noticed that accelerated aging caused an increase in the DPS of TPUs regardless of the aging method applied.

### 2.2. Thermal Properties of Bio-TPUs–Influence—Influence of Aging Factors on the Thermal Stability, Phase Transitionthermal Stability, Phase Transition, and Thermomechanical Behaviorthermo-Mechanical Behavior

**Figure 2:**
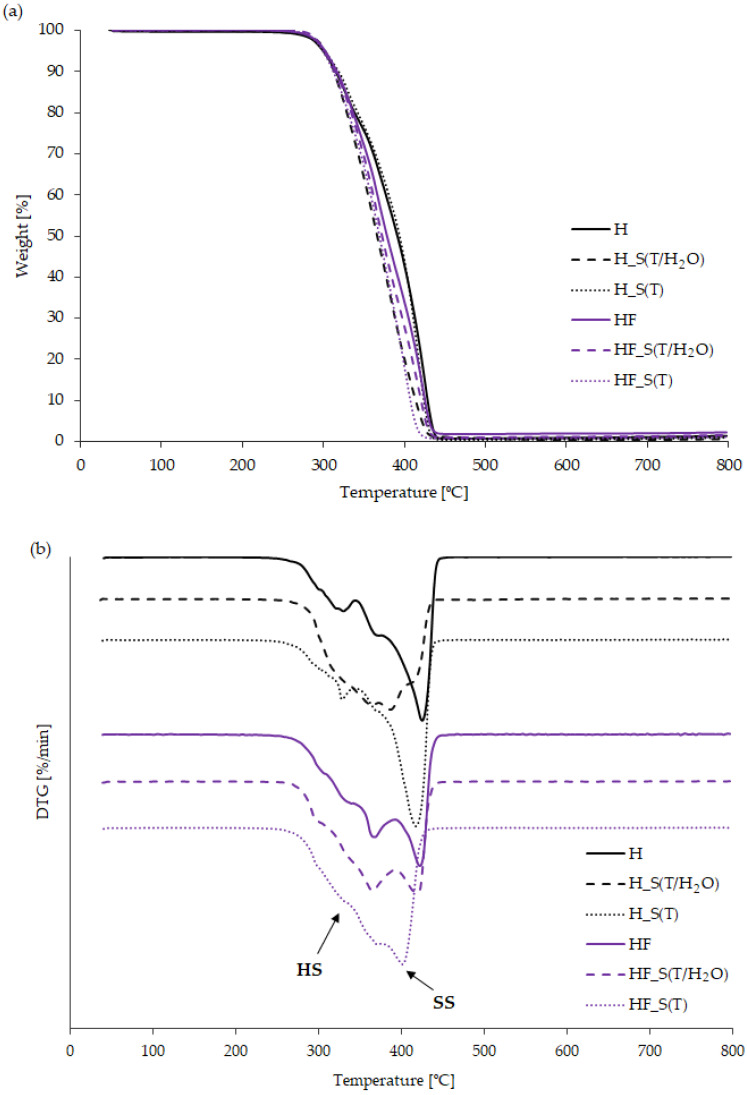
(**a**) TG and (**b**) DTG curves of bio-TPUs before (H and HF) and after accelerated aging (H_S(T/H_2_O; H_S(T), HF_S(T/H_2_O; HF_S(T)).

In order to determine the thermal stability of the reference, modified, and aged bio-TPUs, a thermogravimetric analysis (TGA) was conducted. The results are presented in the thermograms in Figure 2, providing information about the temperature at the onset of the degradation, indicated by a 5 wt.% mass loss (T_5%_). Moreover, the temperatures corresponding to 10% and 50% mass loss (T_10%_ and T_50%_), as well as the temperatures at the maximum thermal degradation rates of the hard and soft segments (T_DTG1_ and T_DTG2_) were determined and are also presented in Table 2.

Based on the TG curves (Figure 2a) and the data presented in Table 2, it is evident that the reference, modified, and aged materials exhibit thermal stability up to approximately 300 °C. The onset temperature of thermal degradation varies among the materials by 1 to 4 °C, regardless of the type or aging method. A slight decrease in T_5%_ was observed in the case of HF materials subjected to both thermal and hydrothermal aging. The thermal stability of the prepared bio-TPUs is comparable to that of polyurethane materials based on HDI diisocyanate, as described in the literature [9]. 

The analysis of DTG curves confirms the typical two-stage thermal degradation process of polyurethane, involving the dissociation of hard segments in the first stage and the degradation of soft segments in the second stage. The phenomenon of thermal degradation is closely related to the phase structure and phase separation in the bio-TPU materials, as observed in the bio-TPUs before the accelerated aging tests. Materials subjected to accelerated aging tests exhibit more complex degradation behavior, with significant changes occurring mainly in the first stage. The degradation rate for each stage depends on both the chemical structure of bio-TPUs and the accelerated aging method. The bio-TPUs in the HF series exhibited a lower degradation rate in the second stage of degradation.

Analyzing Figure 2b, changes in the phase separation of the bio-TPUs subjected to hydrothermal accelerated aging are observable. In the DTG curves, the typical two-stage degradation process is not clearly evident. The modification of the hard segments with partially bio-based isocyanate (HF) resulted in a slightly higher T_5%_. Accelerated aging under both hydrothermal and thermal conditions did not significantly affect T_5%_. However, after thermal accelerated aging, changes in the degradation behavior of the soft segments became evident. According to the literature, polyether-urethanes are known to be more susceptible to thermo-oxidative degradation. Consequently, a decrease in T_DTG2_ by approximately 20 °C was recorded, likely due to chain scission and the formation of oligomeric structures. These changes may lead to reduce thermal stability in the soft segments of bio-TPUs, as soft segments are generally more susceptible to oxidative degradation than hard segments [20].

In the case of thermo-oxidative aging in an aqueous environment, a decrease in T_DTG2_ of soft segments ca. 12 °C was observed for the aged reference material H_S(T/H_2_O). TPUs with modified hard segments (HF_S(T/H_2_O)) showed a decrease in T_DTG2_ of 2 °C. Aging the material at elevated temperatures in an aqueous environment can promote the hydrolysis of urethane and ether bonds. Although ether groups are generally more resistant to hydrolysis than ester groups, they can still undergo hydrolysis under certain conditions, such as high temperatures. As a result, the polyurethane structure weakens, making it more susceptible to thermal degradation. Additionally, water can act as a medium for generating reactive oxygen species, which can initiate oxidation reactions in the polymer, leading to further degradation. An analysis of the residual solid of polyurethane at 800 °C reveals a noticeable decrease in mass after the accelerated aging. Various processes may occur during the polymer degradation, including the breakdown of the polymer chains and the formation of volatile degradation products. These processes likely contribute to the observed changes.

**Figure 3:**
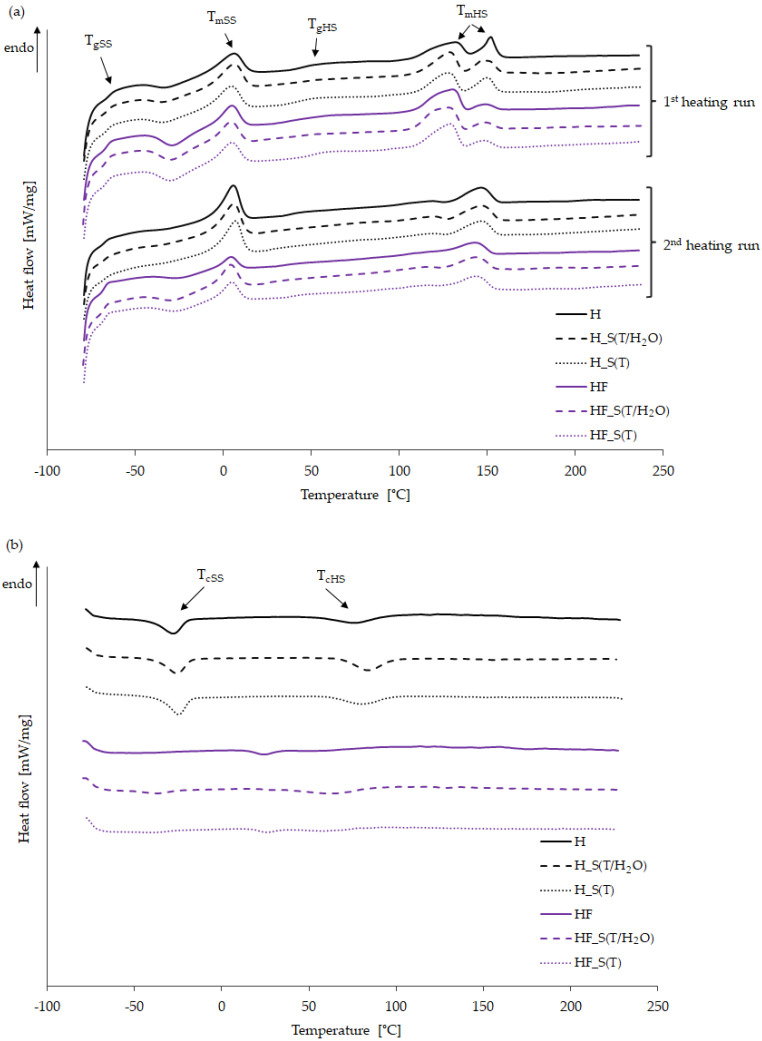
(**a**) The endothermic curves and (**b**) the exothermic curves of bio-TPUs before (H and HF) and after accelerated aging (H_S(T/H_2_O; H_S(T), HF_S(T/H_2_O; HF_S(T)).

Differential scanning calorimetry (DSC) was used to investigate the phase transitions in the bio-TPUs, including the determination of the glass transition temperatures of soft segments (T_gSS_) and hard segments (T_gHS_), as well as the melting and crystallization of SS and HS. Generally, phase transitions corresponding to the hard segments occur at higher temperatures compared to the soft segments [21]. Figure 3 presents both heating and cooling curves. In Figure 3a, curves from the first heating run are shown in the upper part of the chart, while curves from the second heating run are presented in the lower part. It is generally assumed that during the first heating run, the thermal history of the material is erased. Therefore, the discussion of results focuses on the data obtained during cooling and the second heating run. After cooling and the second heating run, single peaks appeared in the endothermic curve in the temperature range corresponding to the melting of hard segments for both bio-TPU materials, including both the reference and modified samples. This observation forms the basis for the subsequent data analysis and discussion.

Considering the glass transition temperature of the soft segments (T_gSS_), it is observed that for all materials, regardless of chemical composition and accelerated aging method, T_gSS_ was ca. −68 °C. When analyzing the melting temperature of the soft segments (T_mSS_) for both the reference and modified bio-TPUs, it was found that this process occurs at the maximum temperature of around 4–5 °C. The higher melting enthalpy of soft segments in the reference materials suggests higher ordering and well-formed soft domains (similar in size and shape) compared to the modified bio-TPUs. Generally, the polyol PO3G used in the synthesis has a linear structure, which tends to favor crystallization. Accelerated aging affects the melting enthalpy of soft segments in the HF series of bio-TPUs, indicating improved phase ordering and an increase in crystallinity [22]. There are two main factors that could contribute to an increase in the degree of crystallinity of the aged HF series. Aging in a high-temperature environment allows molecular chains to rearrange and reorganize, leading to the growth of larger spherulites. On the other hand, the aging process can lead to the re-alignment of broken chains (due to chain scission) into a more ordered structure within the amorphous regions of the semicrystalline polymer [23]. An examination of the exothermic curves revealed that the crystallization of soft segments (T_cSS_) occurs below 0 °C, although it is only visible for the HF series of materials. This could be due to the modification of hard segments affecting the phase mixing and limiting the ordering of the soft phase. Alternatively, it may be attributed to partial degradation occurring in the soft phase.

Above room temperature, the glass transition of hard segments (T_gHS_) was registered in the range of 34 to 63.2 °C, with higher values noted for bio-TPUs with modified hard segments. Accelerated aging under thermal and hydrothermal conditions leads to a decrease in T_gHS_ for the HF series of TPUs. The melting behavior of the hard segments (T_mHS_) shows a slight dependence on their chemical structure, as well as aging method (see Table 3). Interestingly, both parameters play a crucial role in the crystallization of hard segments in bio-TPUs. A modification of HS with partially bio-based isocyanate caused a decrease in crystallization temperature, which may be related to the higher molecular weight of the HS. However, no clear trend was observed regarding the impact of accelerated aging. In general, the use of partially bio-based diisocyanate in bio-TPU synthesis results in a slight decrease in the melting temperature of both soft and hard segments. This phenomenon was also noted in our earlier works [24,25]. In the case of bio-TPUs subjected to accelerated aging, the same trend was registered.

**Figure 4:**
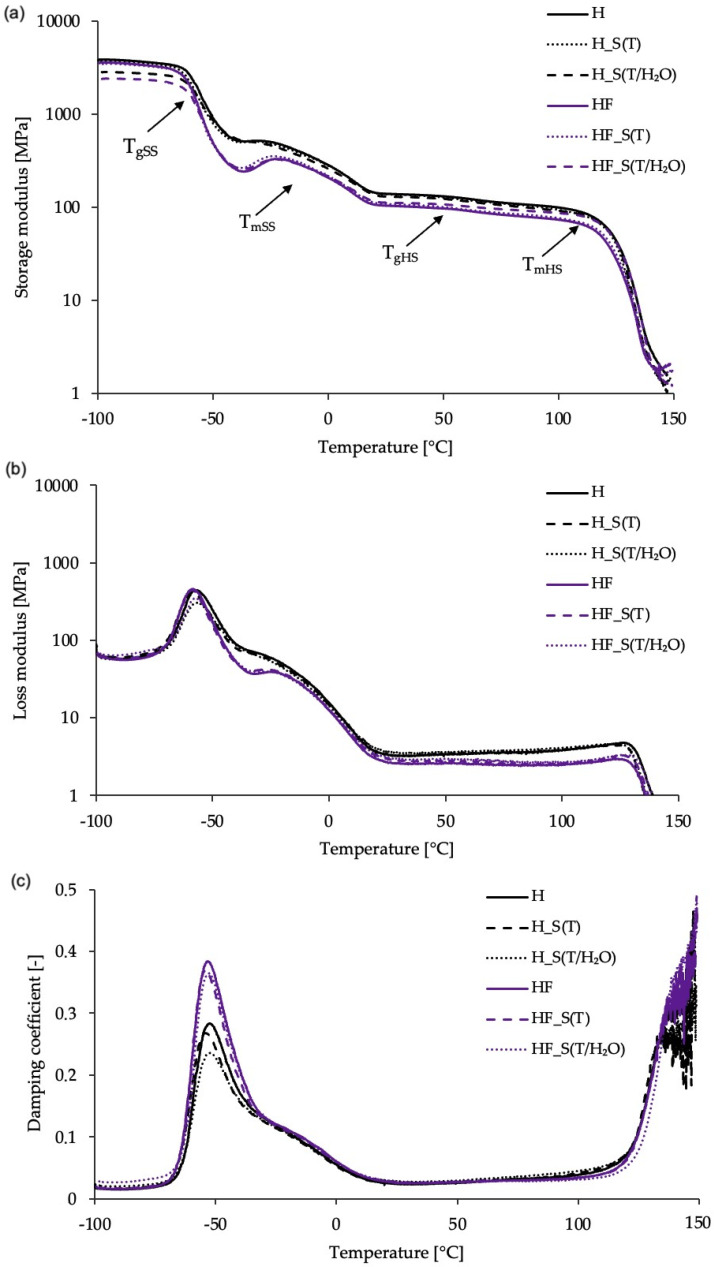
Temperature dependence of (**a**) storage modulus, (**b**) loss modulus, and (**c**) tanδ of bio-TPUs before (H and HF) and after accelerating aging (H_S(T/H_2_O; H_S(T), HF_S(T/H_2_O; HF_S(T)).

The thermo-mechanical properties of the bio-TPU materials, both prior to and after accelerating aging, were investigated under the dynamic condition using the dynamic mechanical thermal analysis (DMTA) method. This technique allows for the study of the viscoelastic behavior of polymers and the determination of the storage modulus (E’), loss modulus (E”), and damping factor (tan δ). The obtained results are presented in Figure 4 and Table 4.

Analyzing Figure 4a, the typical behavior of the storage modulus (E’) vs. the temperature curves for thermoplastic polyurethane elastomers might be observed. In the temperature range from −70 to −40 °C, the glass transition of the soft segments is observed as a sharp drop in the E’ curve. Above this temperature, the melting of soft segments appears as a broad arm in the temperature range of −25 to 15 °C. In the case of bio-TPUs with modified hard segments, the melting of soft segments is preceded by cold crystallization (visible slight increasing in E’). This behavior is observed in HF-based materials, both before and after accelerated aging.

At room temperature, a higher storage modulus was observed for the refence bio-TPUs. Accelerated aging under thermal and hydrothermal conditions caused a decrease in E’ (at 25 °C) for the reference bio-TPUs, while the modified bio-TPUs showed the opposite trend. Furthermore, accelerated aging under hydrothermal conditions resulted in more pronounced changes. This suggests that the degradation mechanisms differed between the two materials. For the reference bio-TPUs, water acted as a plasticizer, leading to decreased stiffness. Analyzing the bio-TPUs with modified hard segments, the slight increase in E’ observed in the aged materials might be the result of chain scissions and crosslinking of the degradation products. Regarding thermal aging, where high temperature is the primary degradation factor, minimal autooxidation was observed regardless of the bio-TPU composition. It is worth mentioning that urethane groups exhibit greater resistance to oxidation compared to the macrodiol building blocks [11]. The glass transition of hard segments was revealed at a temperature ca. 50 °C, regardless of the bio-TPU type. Above 100 °C, the melting of hard segments was observed for all materials.

As mentioned, the impact of incorporating bio-based isocyanate into the structure of TPUs was noted. Compared to the reference material, the modified bio-TPUs exhibited lower stiffness at both at low and room temperatures (see Table 4). This indicates that less energy might be stored by the material. On the other hand, the modification of the hard segments improved the energy dissipation. The modification of the hard segment of bio-TPUs by incorporing partially bio-based isocyanate into teir structure enhances the damping properties of the synthesized materials. This is evidenced by the higher values (Table 4).

## 3. Materials and Methods

### 3.1. Materials

The used materials were poly(trimethylene ether) glycol, with the trade name Velvetol H 2000 (Allessa GmbH, Frankfurt, Germany); hexamethylene diisocyanate (HDI) (Vencorex, Saint-Priest, France); polyisocyanate Tolonate^TM^ X Flo 100 (Vencorex, Saint-Priest, France); 1,3-propanediol Susterra^®^ Propanodiol (DuPont Tate and Lyle Bio Products, Wilmington, DE, USA); and dibutyltin dilaurate (DBTDL) (Sigma Aldrich, Poznań, Poland, division).

The auxiliary chemicals used for the determination of free NCO content were dibutylamine (Sigma Aldrich, Poland division), chlorobenzene (POCH, Gliwice, Poland), acetone (POCH, Gliwice, Poland), 0.1M hydrochloric acid (POCH, Gliwice, Poland), and bromophenol blue (POCH, Gliwice, Poland division).

### 3.2. TPU Preparation and Accelerated Aging

**Figure 5:**
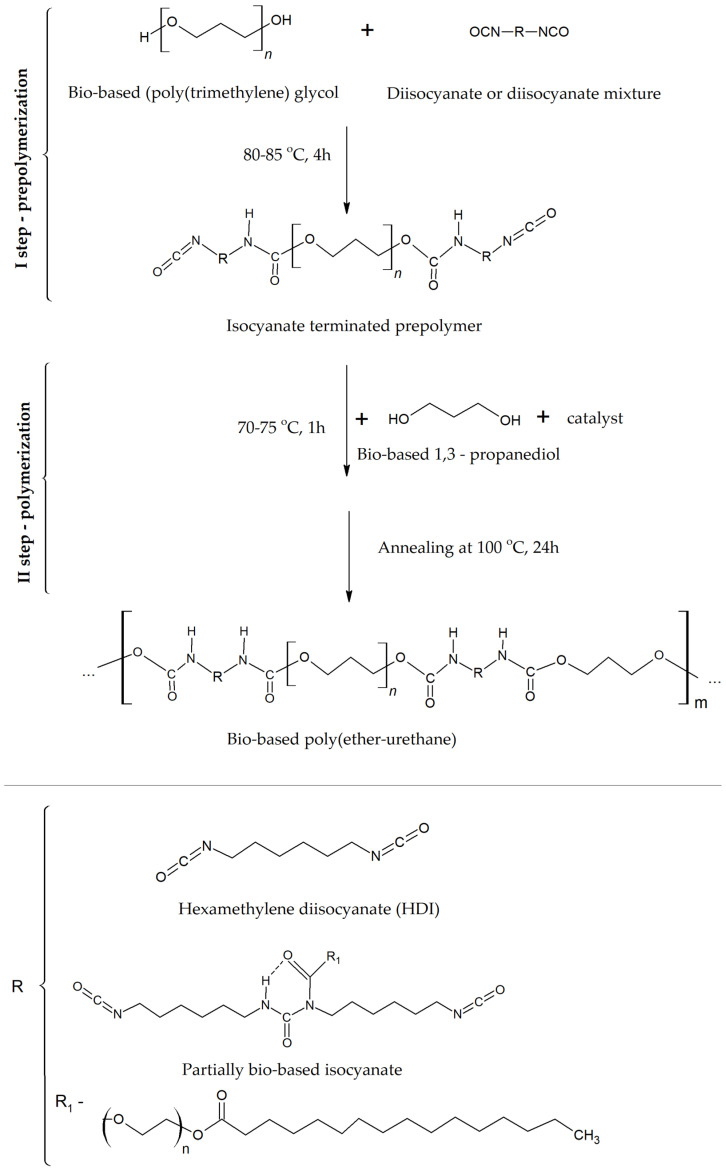
Scheme of the synthesis of bio-TPUs obtained via the prepolymer method.

Bio-TPUs were synthesized using the prepolymer method according to the procedure in [24]. In the first step, a prepolymer was synthesized using polyether polyol, which was reacted with hexamethylene diisocyanate or a diisocyanate mixture based on HDI and partially bio-based polyisocyanate. The polyisocyanate mixture (HF) was composed of 80% HDI (coded as H) and 20% Tolonate^TM^ X Flo 100 (coded as F). For excess NCO groups, the free NCO content was determined using the titration method according to ASTM D 2572-97 and equaled ca. 8%. Next, each prepolymer was extended by a low-molecular-weight chain extender, bio-based 1,3-propanediol, in the presence of dibutyltin dilaurate as a catalyst. All bio-TPU samples were prepared at an equimolar ratio of isocyanate group to hydroxyl group. The molded bio-TPUs were annealed at 100 °C for 24 h in a laboratory oven to obtain fully reacted materials. The reaction scheme is shown in Figure 5.

Bio-TPUs were subjected to the accelerated aging under both thermal and hydrothermal conditions. In the case of the thermal accelerated aging test, the materials were exposed to a temperature of 80 °C for 7 days. The samples were placed in a laboratory oven equipped with air circulation.

The hydrothermal aging test was also conducted in the laboratory oven equipped with air circulation. The samples were immersed in distilled water for 7 days at 70 °C.

The prepared samples (both the reference and aged samples) were coded as follows:

H—bio-TPUs based on only diisocyanate HDI—reference materials;

HF—bio-TPUs based on the diisocyanate mixture—materials with modified hard segments;

H_S(T) and HF_S(T)—the reference and modified bio-TPUs subjected to thermal accelerated aging;

H_S(T/H_2_O) and HF_ S(T/H_2_O)—the reference and modified bio-TPUs subjected to hydrothermal accelerated aging.

### 3.3. Measurements

Fourier transform infrared spectroscopy (FTIR) measurements were conducted using an Invenio-R spectrophotometer (Bruker, Poland division). The measurements were taken in the spectral range of 400–4000 cm^−1^, with 64 scans at a resolution of 2 cm^−1^. An integrated ATR accessory with a single reflection diamond crystal and a DTGS detector was used for the measurements.

Based on the decomposition of normalized FTIR spectra of the carbonyl peak using the FITYK software (1.3.1.), each peak was decomposed into four component peaks. The carbonyl index and the degree of phase separation were calculated using Equations (1) and (2).

(1) $$R=\frac{A_{H\text{-}bonded}}{A_{free}}$$

(2) $$DPS=\frac{R}{R+1}$$

where R—carbonyl hydrogen bonding index; A_H-bonded_—the sum of the peak area corresponding to hydrogen-bonded carbonyl groups; A_free_—the sum of the peak area corresponding to the free carbonyl; DPS—degree of phase separation.

The thermal stability analysis of the materials was carried out using a thermogravimetric analysis (TGA) with a TG 209F3 thermobalance (NETZSCH, Selb, Germany). The measurements were performed under a nitrogen atmosphere in the temperature range of 35 to 800°C, with a heating rate of 10 °C/min.

The dynamic mechanical thermal analysis (DMTA) was conducted using a DMA Q800 analyzer (TA Instruments, New Castle, DE, USA). The measurements were performed in the temperature range of −100 to 150 °C, using a three-point bending mode, with an operating frequency of 10 Hz and a heating rate of 3°C/min.

The differential scanning calorimetry (DSC) measurements were performed using a DSC 204 F1 Phoenix calorimeter (NETZSCH, Selb, Germany) The measurements were taken under an inert atmosphere, with the nitrogen flow rate of 20 mL/min. Each sample, weighing approximately 10 mg, underwent two heating cycles in the range of −80 to 240 °C at a rate of 10 °C/min and one cooling cycle from 240 to −80 °C at a rate of 5 °C/min.

## 4. Conclusions

Gaining knowledge of the influence of different aging factors on bio-based thermoplastic polyurethane elastomers is crucial in light of bio-TPUs’ further applications and to avoid their accumulation in the environment. Nowadays, sustainable materials should be synthesized using bio-based monomers, which was achieved in this work. Incorporating partially bio-based diisocyanate into the structure enhanced the thermal stability and damping properties of the bio-TPUs. Based on the FTIR results and the calculated degree of phase separation, it can be concluded that changes in the materials due to modification or aging occurred at the supramolecular level. Accelerated aging under thermal conditions resulted in a decrease in the T_DTG2_ parameter, reflecting degradation in the range of the soft segments, as predicted. Modification of the hard segments increased the degradation rate of the soft segments and decreased the temperature at maximum degradation. Overall, T_5%_ changed only by ca. 1%. The effect of the application in bio-TPU synthesis with partially bio-based diisocyanate was observed in the phase transition and melting/crystallization behavior of the bio-TPUs, with a slight decrease in the melting temperature for both the soft and hard segments. This phenomenon was also noted in the bio-TPUs subjected to accelerated aging. To sum up, the obtained results provide promising data for planning further modifications of the bio-TPU chemical structure, as well as experiments related to accelerated aging. Nevertheless, the synthesized bio-TPUs in their current form could have numerous potential applications in everyday life, such as in phone cases, watch bands, and other similar items.

## Figures and Tables

**Table 1:** Carbonyl index (R) and degree of phase separation (DPS) calculated based on FTIR spectra in the range of stretching vibration of carbonyl group.

Sample	R	DPS
H	0.83	0.45
H_S(T/H_2_O)	1.50	0.60
H_S(T)	1.49	0.60
HF	0.74	0.42
HF_S(T/H_2_O)	1.31	0.57
HF_S(T)	1.52	0.60

**Table 2:** The thermal properties of the reference, modified, and gained bio-TPUs determined based on TGA.

Sample	Temperature [°C]					Residue at 800 °C [%]
	T_5%_	T_10%_	T_50%_	T_DTG1_	T_DTG2_	
H	300	316	392	329	424	1.40
H_S(T/H_2_O)	303	314	367	364	387	0.52
H_S(T)	301	319	394	329	418	0.95
HF	302	316	378	368	423	2.14
HF_S(T/H_2_O)	300	314	372	367	421	1.65
HF_S(T)	299	312	369	370	401	1.17

**Table 3:** Thermal properties of the reference, modified, and aged bio-TPUs determined from the second heating run using the DSC technique.

Sample	T_Gss_ [°C]	T_mSS_ [°C]	ΔH_mSS_ [J/g]	T_cSS_ [°C]	ΔH_mSS_ [J/g]	T_gHS_ [°C]	T_mHS_ [°C]	ΔH_mHS_ [J/g]	T_cHS_ [°C]	ΔH_cHS_ [J/g]
H	−67	5.4	26.0	−27.7	−67.3	34.0	147	17.5	76.9	−15.0
H_S(T/H_2_O)	−68	5.7	27.2	−25.8	−67.7	44.3	147	17.7	84.0	−18.2
H_S(T)	−68	6.5	25.8	−24.8	−67.7	34.0	147	14.7	80.2	−14.2
HF	−68	4.2	7.8	-	-	63.2	143	14.1	25.9	−3.7
HF_S(T/H_2_O)	−68	4.2	12.8	−37.1	−3.7	42.7	143	15.0	63.7	−8.2
HF_S(T)	−67	4.4	12.3	-	-	48,7	144	16.9	25.8	−10.1

**Table 4:** Thermo-mechanical properties of the reference, modified and aged bio-TPUs.

Sample	E’ [MPa]	E’ at 25 °C [MPa]	E” [MPa]	Tan δ [-]	T_gSS_ [°C]
H	3857	139	441	0.284	−52.5
H_S(T/H_2_O)	2837	130	305	0.236	−52.4
H_S(T)	3780	137	411	0.268	−54.2
HF	3589	104	458	0.384	−53.3
HF_S(T/H_2_O)	2853	112	349	0.365	−52.8
HF_S(T)	3485	108	441	0.369	−53.7

## Data Availability

All data generated or analysed during this study are included in this published article.

## References

[1] Binda G., Kalčíková G., Allan I.J., Hurley R., Rødland E., Spanu D., Nizzetto L. (2024). Microplastic aging processes: Environmental relevance and analytical implications. TrAC-Trends Anal. Chem..

[2] White J.R. (2006). Polymer ageing: Physics, chemistry or engineering? Time to reflect. Comptes Rendus Chim..

[3] Starkova O., Gagani A.I., Karl C.W., Rocha I.B.C.M., Burlakovs J., Krauklis A.E. (2022). Modelling of environmental ageing of polymers and polymer composites—Durability prediction methods. Polymers.

[4] Mouren A., Avérous L. (2023). Aromatic thermoplastic polyurethanes synthesized from different potential sustainable resources. Eur. Polym. J..

[5] Kasprzyk P., Głowińska E., Parcheta-Szwindowska P., Rohde K., Datta J. (2021). Green TPUs from prepolymer mixtures designed by controlling the chemical structure of flexible segments. Int. J. Mol. Sci..

[6] Kasprzyk P., Głowińska E., Datta J. (2021). Structure and properties comparison of poly(ether-urethane)s based on nonpetrochemical and petrochemical polyols obtained by solvent free two-step method. Eur. Polym. J..

[7] Baysal G., Aydın H., Hoşgören H., Uzan S., Karaer H. (2016). Improvement of Synthesis and Dielectric Properties of Polyurethane/Mt-QASs+ (Novel Synthesis). J. Polym. Environ..

[8] Gallu R., Méchin F., Dalmas F., Gérard J.F., Perrin R., Loup F. (2020). On the use of solubility parameters to investigate phase separation-morphology-mechanical behavior relationships of TPU. Polymer.

[9] Głowińska E., Smorawska J., Niesiobędzka J., Datta J. (2024). Structure versus hydrolytic and thermal stability of bio-based thermoplastic polyurethane elastomers composed of hard and soft building blocks with high content of green carbon. J. Therm. Anal. Calorim..

[10] Gopalakrishnan S., Linda Fernando T. (2011). Studies on ageing performance of some novel polyurethanes. J. Chem. Pharm. Res..

[11] Xie F., Zhang T., Bryant P., Kurusingal V., Colwell J.M., Laycock B. (2019). Degradation and stabilization of polyurethane elastomers. Prog. Polym. Sci..

[12] Boubakri A., Haddar N., Elleuch K., Bienvenu Y. (2011). Influence of thermal aging on tensile and creep behavior of thermoplastic polyurethane. Comptes Rendus-Mec..

[13] Rosu L., Varganici C.D., Rosu D., Oprea S. (2021). Effect of thermal aging on the physico-chemical and optical properties of poly(Ester urethane) elastomers designed for passive damping (pads) of the railway. Polymers.

[14] Boubakri A., Elleuch K., Guermazi N., Ayedi H.F. (2009). Investigations on hygrothermal aging of thermoplastic polyurethane material. Mater. Des..

[15] Hong S., Park N.Y., Ju S., Lee A., Shin Y., Kim J.S., Um M.K., Yi J.W., Chae H.G., Park T. (2023). Molecular degradation mechanism of segmented polyurethane and life prediction through accelerated aging test. Polym. Test..

[16] Jana R.N., Bhunia H. (2010). Accelerated hygrothermal and UV aging of thermoplastic polyurethanes. High Perform. Polym..

[17] Bistričić L., Baranović G., Leskovac M., Bajsić E.G. (2010). Hydrogen bonding and mechanical properties of thin films of polyether-based polyurethane-silica nanocomposites. Eur. Polym. J..

[18] Kasprzyk P., Datta J. (2019). Novel bio-based thermoplastic poly(ether-urethane)s. Correlations between the structure, processing and properties. Polymer.

[19] Kasprzyk P., Błażek K., Parcheta P., Datta J. (2020). Green thermoplastic poly(ether-urethane)s–synthesis, chemical structure and selected properties investigation. Polimery/Polymers.

[20] Allan D., Daly J.H., Liggat J.J. (2019). Oxidative and non-oxidative degradation of a TDI-based polyurethane foam: Volatile product and condensed phase characterisation by FTIR and solid state 13 C NMR spectroscopy. Polym. Degrad. Stab..

[21] Datta J., Kasprzyk P. (2018). Thermoplastic polyurethanes derived from petrochemical or renewable resources: A comprehensive review. Polym. Eng. Sci..

[22] Saiani A., Novak A., Rodier L., Eeckhaut G., Leenslag J.W., Higgins J.S. (2007). Origin of Multiple Melting Endotherms in a High Hard Block Content Polyurethane. 1. Thermodynamic Investigation. Macromolecules.

[23] Chang B.P., Mohanty A.K., Misra M. (2020). Studies on durability of sustainable biobased composites: A review. RSC Adv..

[24] Smorawska J., Wloch M., Głowińska E. (2022). Structure-property relationship, and multiple processing studies of novel bio-based thermoplastic polyurethane elastomers. Materials.

[25] Głowińska E., Kasprzyk P., Datta J. (2021). The green approach to the synthesis of bio-based thermoplastic polyurethane elastomers with partially bio-based hard blocks. Materials.

